# IRIS3: integrated cell-type-specific regulon inference server from single-cell RNA-Seq

**DOI:** 10.1093/nar/gkaa394

**Published:** 2020-05-18

**Authors:** Anjun Ma, Cankun Wang, Yuzhou Chang, Faith H Brennan, Adam McDermaid, Bingqiang Liu, Chi Zhang, Phillip G Popovich, Qin Ma

**Affiliations:** Department of Biomedical Informatics, College of Medicine, The Ohio State University, Columbus, OH 43210, USA; Department of Biomedical Informatics, College of Medicine, The Ohio State University, Columbus, OH 43210, USA; Department of Biomedical Informatics, College of Medicine, The Ohio State University, Columbus, OH 43210, USA; Department of Neuroscience, Center for Brain and Spinal Cord Repair, Belford Center for Spinal Cord Injury, The Ohio State University Wexner Medical Center, Columbus, OH 43210, USA; Imagenetics, Sanford Health, Sioux Falls, SD 57104, USA; Department of Internal Medicine, Sanford School of Medicine, University of South Dakota, Vermillion, SD 57069, USA; School of Mathematics, Shandong University, Jinan 250100, China; Department of Medical & Molecular Genetics, Indiana University, School of Medicine, Indianapolis, IN 46202, USA; Department of Neuroscience, Center for Brain and Spinal Cord Repair, Belford Center for Spinal Cord Injury, The Ohio State University Wexner Medical Center, Columbus, OH 43210, USA; Department of Biomedical Informatics, College of Medicine, The Ohio State University, Columbus, OH 43210, USA

## Abstract

A group of genes controlled as a unit, usually by the same repressor or activator gene, is known as a regulon. The ability to identify active regulons within a specific cell type, i.e., cell-type-specific regulons (CTSR), provides an extraordinary opportunity to pinpoint crucial regulators and target genes responsible for complex diseases. However, the identification of CTSRs from single-cell RNA-Seq (scRNA-Seq) data is computationally challenging. We introduce IRIS3, the first-of-its-kind web server for CTSR inference from scRNA-Seq data for human and mouse. IRIS3 is an easy-to-use server empowered by over 20 functionalities to support comprehensive interpretations and graphical visualizations of identified CTSRs. CTSR data can be used to reliably characterize and distinguish the corresponding cell type from others and can be combined with other computational or experimental analyses for biomedical studies. CTSRs can, therefore, aid in the discovery of major regulatory mechanisms and allow reliable constructions of global transcriptional regulation networks encoded in a specific cell type. The broader impact of IRIS3 includes, but is not limited to, investigation of complex diseases hierarchies and heterogeneity, causal gene regulatory network construction, and drug development. IRIS3 is freely accessible from https://bmbl.bmi.osumc.edu/iris3/ with no login requirement.

## BACKGROUND

Sophisticated gene regulatory mechanisms define and maintain transcriptional states, and in turn, these diverse states influence the heterogeneous cellular functions in different cell types (1). Within a global gene regulatory system, a regulon represents a maximal group of genes co-regulated by the same transcription factors (**TFs**). A clear assessment and annotation of regulons and the TFs that control them is an effective strategy to pinpoint crucial and heterogeneous regulatory mechanisms encoded in diverse cell types, and those responsible for the development of diseases (2,3).

In the past decade, several computational tools have been developed to identify regulons in human and mouse using bulk RNA-Sequencing (**RNA-Seq**) data, e.g. iRegulon (4) and Onco-regulon (5). However, bulk tissue RNA-Seq data only enables the prediction of regulons at sample/patient levels, with the assumption that cells maintain the same regulatory mechanisms across diverse cell types. In addition, these tools rely heavily on prior knowledge of benchmarked connections between TFs and their target genes. As a result, single-cell RNA-Seq (**scRNA-Seq**) technologies have rapidly developed. Massive repositories of scRNA-Seq data in the past five years provide an unprecedented opportunity to predict regulons that are specifically active in heterogeneous cell types and during transitions between different cell types (6). In 2017, Aibar *et al.* developed SCENIC to identify regulons and construct gene regulatory networks from scRNA-Seq data (7). Using SCENIC, Rambow *et al.* found that the Retinoid X Receptor signaling is promising for the relapse in melanoma, and proposed a potential therapy for delaying the development of drug resistance by blocking the signal-related regulons exhibited in neural crest stem cells (8); Kristofer *et al.* built a single-cell transcriptional and TF-regulon atlas revealing the regulatory heterogeneity of different cell types in the aging Drosophila brain (9); Suo *et al.* created a mouse cell atlas containing 8,461 genes in 61 637 cells sampled from 98 cell types across 40 organs (10). They identified 202 cell type activated regulons and essential regulators which serve as valuable resources for the broad biological community.

Not surprisingly, the successful identification of regulons at the single-cell level can improve the detection of heterogeneous transcriptional regulatory mechanisms across various cell types and allows for reliable constructions of global gene regulatory networks encoded in complex diseases. Hence, it is critical to study cell-type-specific regulons (**CTSRs**). A CTSR is a group of genes co-regulated by the same TF within a specific cell type, and therefore shares the same *cis*-regulatory motif (**motif**) of the underlying TF. However, current limitations for CTSR identification include: (i) existing tools focus on inferring regulons in static cell types or given cell types and ignore the dynamic changes of the gene regulatory mechanisms across different cell types; (ii) *de novo* motif prediction has not been organically integrated into existing tools, giving rise to limited power in predicting novel regulons that are not documented in the literature and (iii) these tools require substantial programming skills in practical applications and are not suitable for the scientists without systematic computational training. Hence, gaps still exist in identifying meaningful CTSRs and no user-friendly web servers are available to identify CTSRs from scRNA-Seq data. All these challenges drive the need to develop an easy-to-use and effective tool for CTSR identification.

In this study, we developed the first-of-its-kind web server for CTSR inference from human or mouse scRNA-Seq data, named **IRIS3** (Integrative Cell-type-specific Regulon Inference Server from Single-cell RNA-Seq). It is streamlined by a seamless integration of multiple widely-used tools, e.g., DrImpute (11), scran (12), Seurat (13), QUBIC2 (14), DMINDA2.0 (15) and MEME (16). Specifically, there are four unique features in the IRIS3 framework: (i) it is an all-in-one framework for CTSR identification, incorporating biclustering for cell-type-specific gene module detection and *de novo* motif prediction for potential novel regulons discovery; (ii) it provides informative CTSR interpretations in support of the in-depth analysis of heterogeneous regulatory mechanisms; (iii) it is equipped with a user-friendly web interface that requires no programming knowledge, with a simple submission process, comprehensive scRNA-Seq data analysis functionalities, and highly-interactive visualizations and (iv) it substantially improves the identification of novel regulatory mechanisms compared to current tools, and allows reliable constructions of global transcriptional regulation networks encoded in a specific cell type.

We used 19 scRNA-Seq datasets to benchmark IRIS3, in terms of the motif specificity and cell type specificity of the identified CTSR, number of differentially expressed genes (**DEGs**) covered by CTSRs, and the biological meaning of predicted CTSRs. IRIS3 demonstrated superior performance compared to the widely used tool, SCENIC, in CTSR identification from the benchmark datasets. Moreover, another 27 datasets (including 49–8522 cells, 13 10× datasets and 12 different tissues) have been tested by independent users demonstrating the reproducibility and robustness of IRIS3. Overall, our tool provides informative interpretations of all the identified CTSRs with interactive visualizations. We believe that IRIS3 is a highly advantageous and easy-to-use web server for CTSR inference. Finally, IRIS3 has the potential to be integrated with other computational or experimental tools in biomedical research, including but not limited to complex disease hierarchies and heterogeneities, causal gene regulatory network construction, and drug development (17–19).

## OVERALL DESIGN OF THE IRIS3 FRAMEWORK

IRIS3 is an integrated framework and takes scRNA-Seq data as the only input for submission. Compatible files include gene expression matrices (with each row representing a gene, each column representing a cell, and each element representing the expression value of a gene in the corresponding cell) and the standard output folder of Cell Ranger from 10X Genomics (20). Specifically, there are three acceptable formats of the required input: (i) a single .txt or .csv formatted gene expression matrix, (ii) an hdf5 feature barcode matrix or (iii) a 10× unique output folder with three files recording information of barcodes, features, and gene expressions. Compressed files are encouraged to decrease the uploading time. IRIS3 accepted Gene Symbols (e.g. HSPA9), Ensembl Gene IDs (e.g. ENSG00000113013), or Transcript IDs (e.g. ENSMUST00000074805). Human and mouse genes are annotated by using the org.HS.eg.db and org.Mn.eg.db R package, respectively. Once an input file is successfully uploaded, users can specify the species and can change parameter settings, such as turn on/off imputation of scRNA-Seq data. Users also have the option to upload a benchmarked cell type file (with the first column representing cell names and the second column representing cell types), or a gene module list file (one gene list per column) in support of CTSR identification (Supplementary Methods).

**Figure 1.**
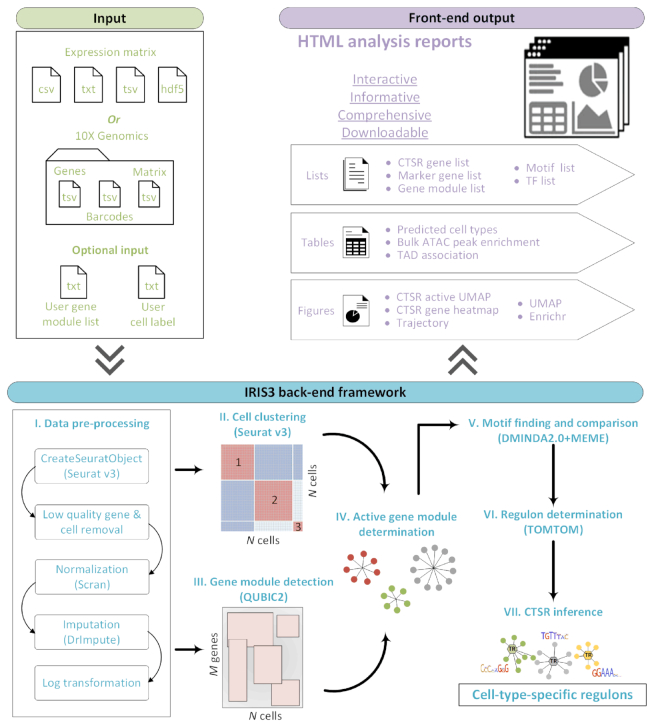
The workflow of IRIS3. The only required input is the scRNA-Seq expression matrix. Seven steps are used to infer CTSRs. A user can upload reference gene modules (lists) for an additional CTSR inference, and the uploaded cell labels can be used as a benchmark for predicted cell type evaluation and substitution for CTSR inference. The output report, along with a unique job ID, will be generated and emailed to the user once the analysis is complete.

A submission screenshot of an example dataset is shown in Supplementary Figure S1. Empowered by the *de novo* motif finding function, IRIS3 can identify putative TFs and TF-gene interactions for a specific cell type. However, the *de novo* algorithm is usually time-consuming, especially when a large number of cell-type-specific gene modules are identified (∼10–20 h). Because of this, users can select the fast version to only identify the top 100 gene modules (five times quicker than the default setting), which will identify the top significant CTSRs. In the accelerated mode, fewer CTSRs will be identified but the most significant CTSRs are usually retained. The specific parameter settings for default and fast versions are listed in Supplementary Table S1. Specifically, seven major steps are included (Figure 1):

### Step I: Data pre-processing

The gene expression data is first loaded through the submission page, and a Seurat object is created. Genes with zero values in >99.9% of cells, and cells with less than 200 non-zero expressed genes, are removed to obtain reliable and robust analytical performance (21). Data normalization status is auto-detected by considering integers as non-normalized values, whereas decimals are considered normalized. The unnormalized data will be normalized by scran (12). An optional imputation step is provided on the submission page. Finally, the expression values are log-normalized ${\rm{log}}(x + 1$) to rescale the data.

### Step II: Cell clustering

Cell types are predicted by Seurat (version 3.1), with most of the parameters set to their default values. The default number of principal components is ten, as suggested by the Seurat tutorial, and an elbow plot is generated for each test dataset (22). Normally, the top ten principal components can cover 85–95% of the data variation, which is enough for feature selection. Cells are clustered using the top ten principal components and a clustering resolution of 0.8 (both default values in Seurat). Note that the cell types mentioned in the following sections of this study are referred to as the computationally predicted cell clusters. The output of this step is a two-column cell label that will be used in Step IV and additional trajectory analysis.

### Step III: Gene module detection

The pre-processed gene expression data from *Step I* is analyzed by our in-house biclustering tool, QUBIC2, for gene module detection (Supplementary Method S1). The previous version of QUBIC has been proven to be one of the top-performing methods in capturing a high proportion of biclusters, enriched by functional biological pathways, effectively and efficiently (23,24). We have demonstrated QUBIC2 shows improved performance compared to QUBIC, especially in scRNA-seq analysis (14). Each of the identified biclusters represents a group of co-expressed genes under a specific subset of cells.

### Step IV: Active gene module determination

We consider the component genes of a bicluster responsive to the regulatory signals in a specific cell type if the cells in the bicluster are highly consistent with the cells in the cell type cluster. To determine the consistency, a hypergeometric enrichment test is performed using the cell types predicted from *Step II* (or the uploaded cell types by users) and the cell components of identified biclusters from *Step III*. The *P-*value of a bicluster corresponding to a specific cell type is Bonferroni-adjusted by multiplying ${N_{cell\;type}} \times {N_{bicluster}}$, where ${N_{cell\;type}}$ denotes the number of cell types and ${N_{bicluster}}$ denotes the total number of biclusters. A bicluster is considered to be active in the corresponding cell type if the cell hypergeometric result is significant (adj.*P* < 0.05) (Supplementary Method S2). Genes included in the bicluster are assigned as an active gene module in that cell type.

### Step V: Motif finding and comparison

For each cell type, motifs are identified in each active gene module via *de novo* motif prediction functions in MEME (16) and DMINDA2 (15,25) (Supplementary Methods S3 and S4). The upstream promoter sequences of each gene are extracted (1,000-bp length by default, and are adjustable by users on the submission page) using the hg38/mm10 reference genome. The reference genomes of human and mouse are integrated in the BSgenome.Hsapiens.UCSC.hg38 and BSgenome.Mmusculus.UCSC.mm10 R packages, respectively.

### Step VI: Regulon determination

The identified motifs in a specific cell type are clustered and annotated with the best matching known motifs from the HOCOMOCO database (V11) (26) using TOMTOM (27). Matching motifs are filtered by removing HOCOMOCO targets for those with a *q*-value of greater than 0.05. The *q*-value is the minimal false discovery rate at which the observed similarity would be deemed significant. For each of the motif clusters, the corresponding nonredundant gene list is named as a regulon.

### Step VII: CTSR inference

For each regulon, its regulon activity score (**RAS**) in a cell is calculated based on the rank of the expression value in the cell for all the involved genes. The regulon specificity score (**RSS**) for a cell type can then be calculated according to the entropy of RAS of cells within the cell type compared to other cell types. A RSS ranges from 0 to 1, with a higher value representing greater specificity of a regulon in the cell type. An empirical *P-*value of a regulon's RSS can be estimated by comparing it with the RSSs of randomly selected gene sets (having the same number of genes in this regulon through a bootstrap method) in the same cell type, 10 000 times. Regulon *P-*values are Bonferroni-adjusted by multiplying the number of regulons in the exact cell type. Regulons with adjusted *P-*values <0.05 (by default) are considered CTSRs (see details in the Supplementary Method S5 and Figure S2). We provide an option at the top of the ‘Regulon details’ page, allowing users to choose the significance threshold of the RSS adjusted *P-*value from 0.001, 0.01, 0.05.

A final comprehensive report is generated to support result interpretation, including interactive cell clustering UMAP (28), cell-gene-regulon heatmap, TF and *de novo* motif information, pathway enrichment, ATAC-Seq peak enrichment, topologically-associated-domain coverage, CTSR inference, trajectory analysis, DEGs and regulon RAS UMAP. All integrated tools are listed in Supplementary Table S2.

## PERFORMANCE AND EVALUATION

To evaluate the performance of IRIS3, we compare the predicted CTSRs with those identified by SCENIC from 19 scRNA-Seq datasets (Supplementary Table S3) (8,29–45). These datasets have cell counts between 148 and 5069 (including three 10X Genomics datasets) and were collected from Gene Expression Omnibus and the European Bioinformatics Institute. Results of the 19 datasets can be accessed at (https://bmbl.bmi.osumc.edu/iris3/more.php#6test_data) or searching corresponding job IDs from the main page. The same cell types, either predicted using Seurat or provided by the original paper, were used for CTSR identification in both IRIS3 and SCENIC. Moreover, another 27 datasets (including 49–8522 cells, 13 10× datasets and 12 different tissues) have been tested by independent users demonstrating the reproducibility and robustness of IRIS3 (Supplementary Table S3).

**Figure 2.**
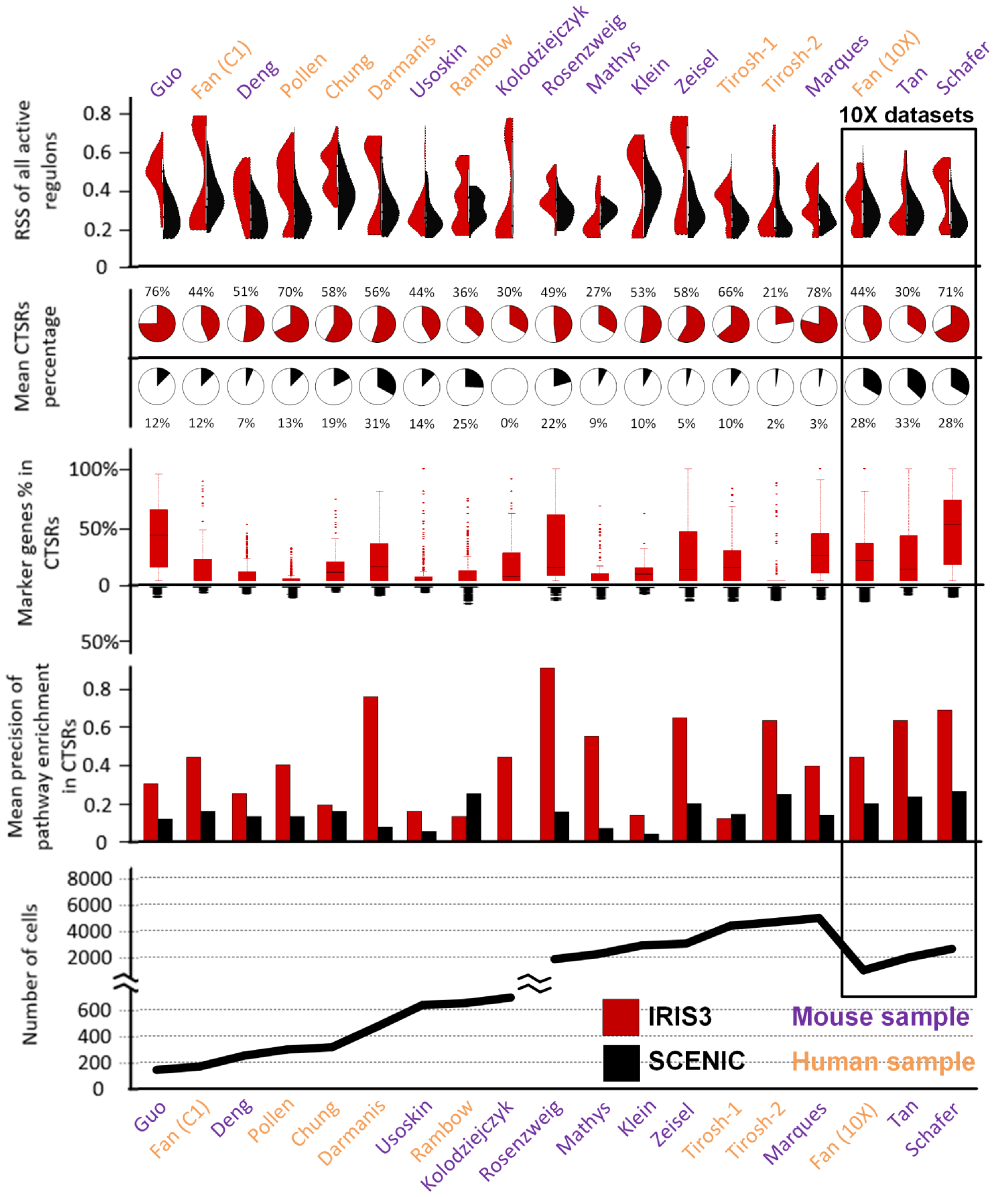
CTSR evaluation and comparison between IRIS3 and SCENIC. All 19 scRNA-Seq datasets (eight human samples in yellow and 11 mouse samples in purple) were tested using the default parameter settings in IRIS3 (red) and SCENIC (black). Note: SCENIC failed to identify any regulons from the dataset by Kolodziejczyk *et al.* as the gene names are not compatible with its embedded database. The data are arranged from left to right in increasing order of cell number, with, 10X datasets grouped and highlighted in the black box to the right. Line one: violin plots of RSS scores in all regulons; Line 2: pie charts of the average percentages of CTSR in regulons per cell type; Line 3: box plots of covered DEGs percentage in each CTSR. We used the same DEGs identified from Seurat for both IRIS3 and SCENIC, and the combination of genes in all SCENIC regulons were used as the denominator since SCENIC was not defining cell-type-specific regulons; Line 4: the mean precision of KEGG pathway enrichment; Line 5: number of cells.

As shown in Figure 2 and Supplementary Table S4, regulons identified by IRIS3 have higher RSSs than SCENIC, indicating that these regulons are more specifically active in the corresponding cell types. Among all 19 datasets, 50.7% of the regulons predicted by IRIS3 are CTSRs per cell type, whereas only 14.8% of the regulons predicted SCENIC are CTSRs. Meanwhile, a CTSR with more DEGs derived from differential expression analysis can be used to define the cell types and generally have a higher RSS. Our analysis suggested the CTSRs identified by IRIS3 are more enriched by DEGs than the ones identified by SCENIC. To assess the biological functions of the CTSRs, we performed pathway enrichment analysis against KEGG pathways by using the Enrichr R package (46). The precision of the enrichment test is the number of KEGG-pathway-enriched CTSRs divided by the total number of CTSRs in a cell type. In 17 out of 19 datasets, IRIS3 achieved significantly higher average precision scores (average precision score: 0.45) than SCENIC (average precision score: 0.16) in all the cell types, indicating that the CTSRs identified by IRIS3 are more biologically meaningful. Overall, IRIS3 shows better performance in identifying CTSRs in terms of effectiveness and biological relevance.

## CASE STUDY OF MOUSE BRAIN CELLS

To illustrate the data analysis and integration functions of IRIS3, we used a mouse dataset, containing 19 972 genes and 3005 cells isolated from the mouse somatosensory cortex and hippocampal CA1 region (40). Seven general cell types (CT) are annotated in the original paper: cells of oligodendrocyte lineage (for simplicity, labeled ‘oligodendrocytes’ in the IRIS server and in Figure 5A), hippocampal (CA1) pyramidal cells, primary somatosensory cortex (SS) pyramidal cells, microglia, interneurons, endothelial and mural cells and astrocytes and ependymal cells. The CT are simply annotated CT1-CT7 in this section. We considered these cell labels as benchmarks and used them for CTSR identification.

### Clustering and differentially expressed genes

**Figure 3.**
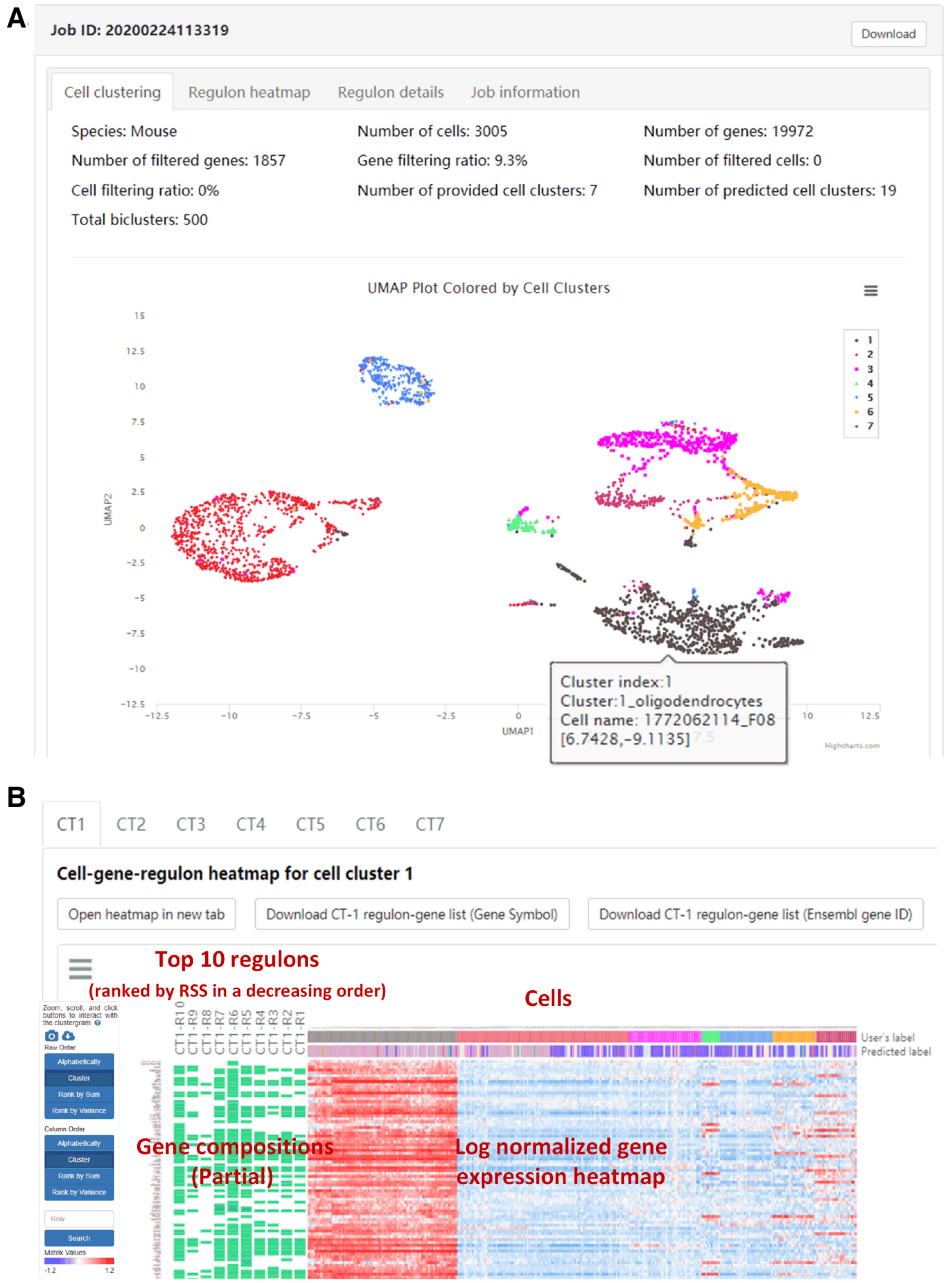
Overall graphical interpretation of cell types and regulons. (**A**) An interactive UMAP is integrated to visualize cell types. Hovering over clusters reveals CT1 includes cells of the oligodendrocyte lineage. The table below shows the overall number of cells and regulons in each cell type. (**B**) The heatmap, empowered by Clustergrammer, showcases the expression pattern of genes from the top ten CTSRs in the corresponding cell type. Users can rearrange the columns and rows by grouping genes in one CTSR or cells in a cell type.

A job ID and a download button are located at the top of the result page of IRIS3 for retrieving detailed and intermediate outcomes (e.g. biclusters, gene modules, etc.). With default parameters for the mouse brain cell dataset, IRIS3 identified 164 regulons, including 95 CTSRs among all seven CTs. We integrated an interactive UMAP visualization to provide a dynamic and clear interpretation of cell types (Figure 3A). The UMAP can be downloaded in diverse formats (e.g. *pdf* and *jpeg*) by clicking on the three-bar symbol at the right corner. The performance of cell clustering is evaluated by comparing the similarity of clustering labels with users’ labels via four indexes: Rand Index, Adjusted Rand Index, Jaccard Index and Fowlkes and Mallows's Index (Supplementary Method S6). The clustering function will be retained, even when users upload a cell type label file. Additional information is provided with separate active buttons to show a high-resolution UMAP, a trajectory plot, the top-100 DEGs in each cluster, a Silhouette plot, and a Sankey plot (Supplementary Figure S3).

### Overall interpretation of regulons from one specific cell type

To aid in the overall interpretation of regulons, we integrate Clustergrammer, an interactive heatmap visualization method (47), to display the cell-gene-regulon heatmap of each cell type (Figure 3B). Both gene compositions of regulons and their expression values across different cell types can be intuitively displayed in such a heatmap. Regulons are ranked in increasing order of the empirical *P-*values of RSS as described above, and a regulon is named as CT*n*-R*m* with *n* representing the index of cell type and *m* represents the regulon rank. Due to space limitations, only the top ten regulons and their corresponding genes are shown in the heatmap, with the component genes of each regulon indicated by green rectangles. The heatmap records the log-transformed expression level of each top-ten-regulon-covered gene across all cells (Supplementary Method S7). Cell names, user-provided cell type labels (if submitted), and cell types labels predicted by Seurat are shown on the heatmap. This heatmap can also be sorted by gene and cell by double-clicking on the appropriate area on the image. Conveniently, a series of gene enrichment tests can be directly performed on the heatmap using the integrated Enrichr function in the Clustergrammer framework. The complete regulon-gene list can be downloaded by clicking on the download buttons above the heatmap (either in gene symbol or Ensembl ID), and users can switch to regulon results in other cell types by clicking on the corresponding labels.

### Comprehensive interpretation of a regulon

**Figure 4.**
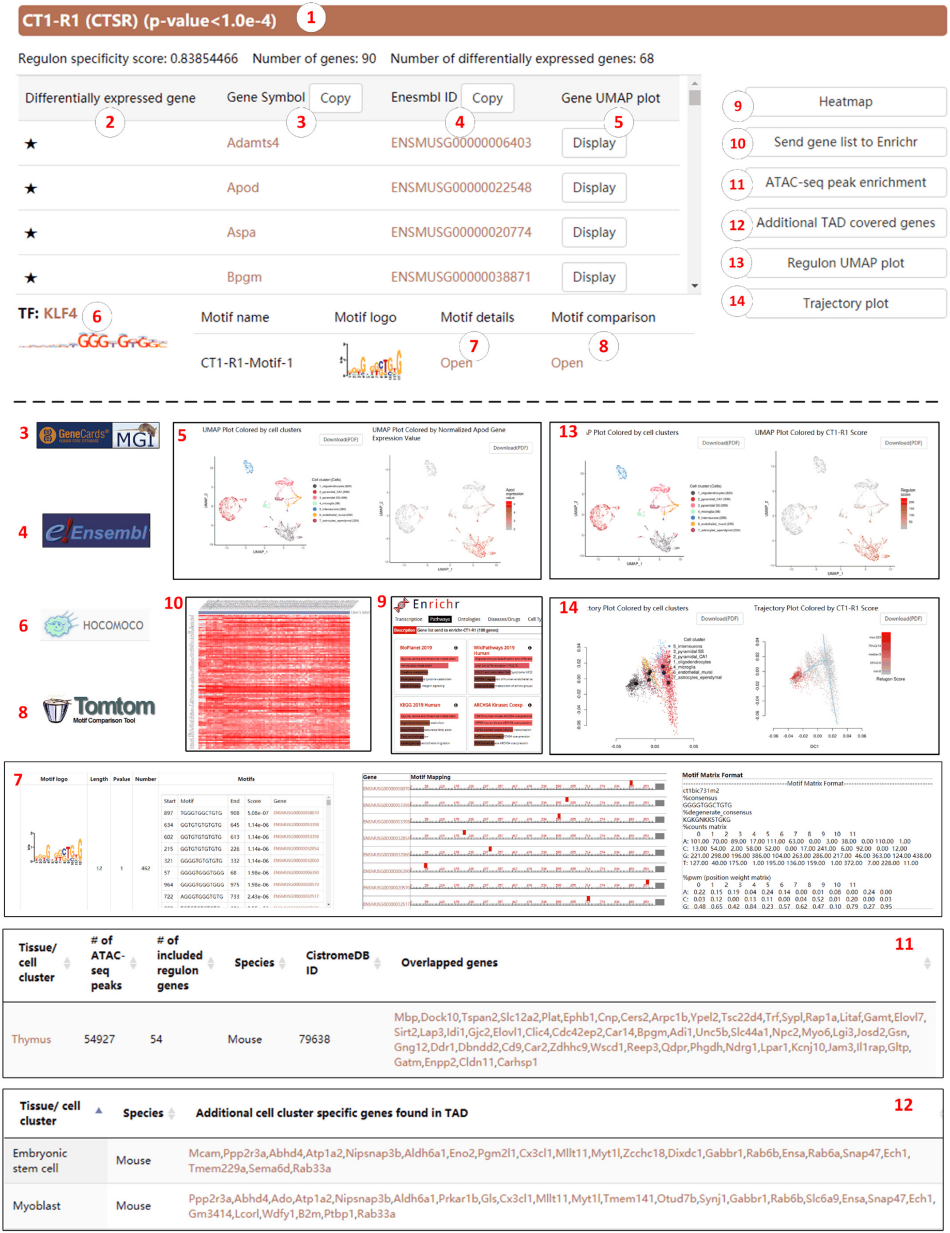
A single regulon interpretation. (1) Each regulon is named by the cell type index and regulon number. A regulon is a cell-type-specific regulon (CTSR) if the adjusted P-value of the regulon specificity score (RSS) is less than 0.05. CTSRs are in orange, and insignificant regulons are in gray. All regulons are ranked in decreasing order of RSS, so that, insignificant regulons are placed behind CTSRs. (2) Stars indicate differentially expressed genes identified in each cluster using Seurat. (3) Gene symbols and links to the GeneCards (Human) or the Mouse Genome Informatics (MGI) website. (4) Corresponding gene Ensembl ID and link to the website. (5) Gene expression UMAP and comparison to the cell types. (6) The corresponding TF with a link to the HOCOMOCO database. (7) Detailed motif finding results including positions, sequences, position weight matrix, etc. (8) Motif details linking to the TOMTOM database. (9) A Clustergrammer heatmap showing the expression values of all genes of this regulon and cell type. (10) The Enrichr link to the enrichment analysis of this regulon. (11) Bulk ATAC peak enrichment test results. (12) Coverage of regulon genes and the topologically associated regions. (13) The regulon activity UMAP and comparison to the cell types. (14) Trajectory analysis colored by cell type and regulon activity, respectively.

IRIS3 provides detailed analyses for each individual regulon to interpret detailed information for the involved genes, motifs, and TF. Taking CT1-R1 (the first regulon in cell type 1) as an example (Figure 4), this regulon includes 90 genes co-regulated by the same TF, KLF4. CT1-R1 is marked as a CTSR based on a significant RSS of 0.84, in which the adjusted empirical *P-*value is less than 1 × 10^−4^. Of all the 90 genes, 68 are differentially expressed in CT1 (marked with stars), according to the differential expression analysis using Seurat. Details of each gene can be found through the corresponding Gene Symbol and Ensembl Gene ID linking the databases, respectively. The gene UMAP indicates the expression distribution of the specific gene on all cells. Three *de novo* identified motif patterns that are conservatively located within the 1000 bp upstream region of all 90 genes are listed and ranked in the increasing order of *P-*values of the motif occurrence randomness. The representative motif shown on the right panel and the interactive motif logo (the 12-bp consensus sequence) can direct users to a detailed motif mapping result page, including the motif *P-*value, related genes, binding site occurrences, and motif position weight matrix. Further motif validations were carried out by comparing the motif sequence occurrences to the TOMTOM database. KLF4 is considered to be the TF regulating these 90 genes due to a significant TF-motif matching *P-*value to CT1-R1-Motif-1, and more information can be found on the HOCOMOCO database by clicking on the TF name or TF logo.

A local Clustergrammer heatmap can be generated by clicking on the ‘Heatmap’ button to display the expression level of all CT1-R1 genes in cells of the oligodendrocyte lineage. Further functional enrichment analyses can be conducted through the enrichment function integrated with the heatmap as described above. Alternatively, users can click the ‘Send gene list to Enrichr’ button to view the complete enrichment results on the Enrichr website. Gene regulation is also related to chromatin availability (ATAC-Seq) and long-distance regulation in topologically associated domains (Hi-C). Due to the limited public availability of single-cell ATAC-Seq and Hi-C, we choose to use bulk-level data to provide soft validation of TF-gene linkages in one regulon. The ‘ATAC-seq peak enrichment’ function will provide feedback on genes included in CT1-R1 whose corresponding chromatin region is also accessible in related ATAC-Seq samplings, ranked by the gene coverage rate in the decreasing order (Supplementary Method S8). Meanwhile, the ‘Additional TAD covered genes’ function traces back potential genes not covered in CT1-R1 but may be topologically co-regulated in oligodendrocytes (Supplementary Method S9). Users can compare the distribution of regulon activity of each cell among cell types using the ‘Regulon UMAP plot’ function. Furthermore, we use Slingshot (48) for cell trajectory inference. The users can access this functionality by clicking on the ‘Trajectory plot’ button and compare the trajectory path to RAS distributions.

### Biological relevance of CTSRs

**Figure 5.**
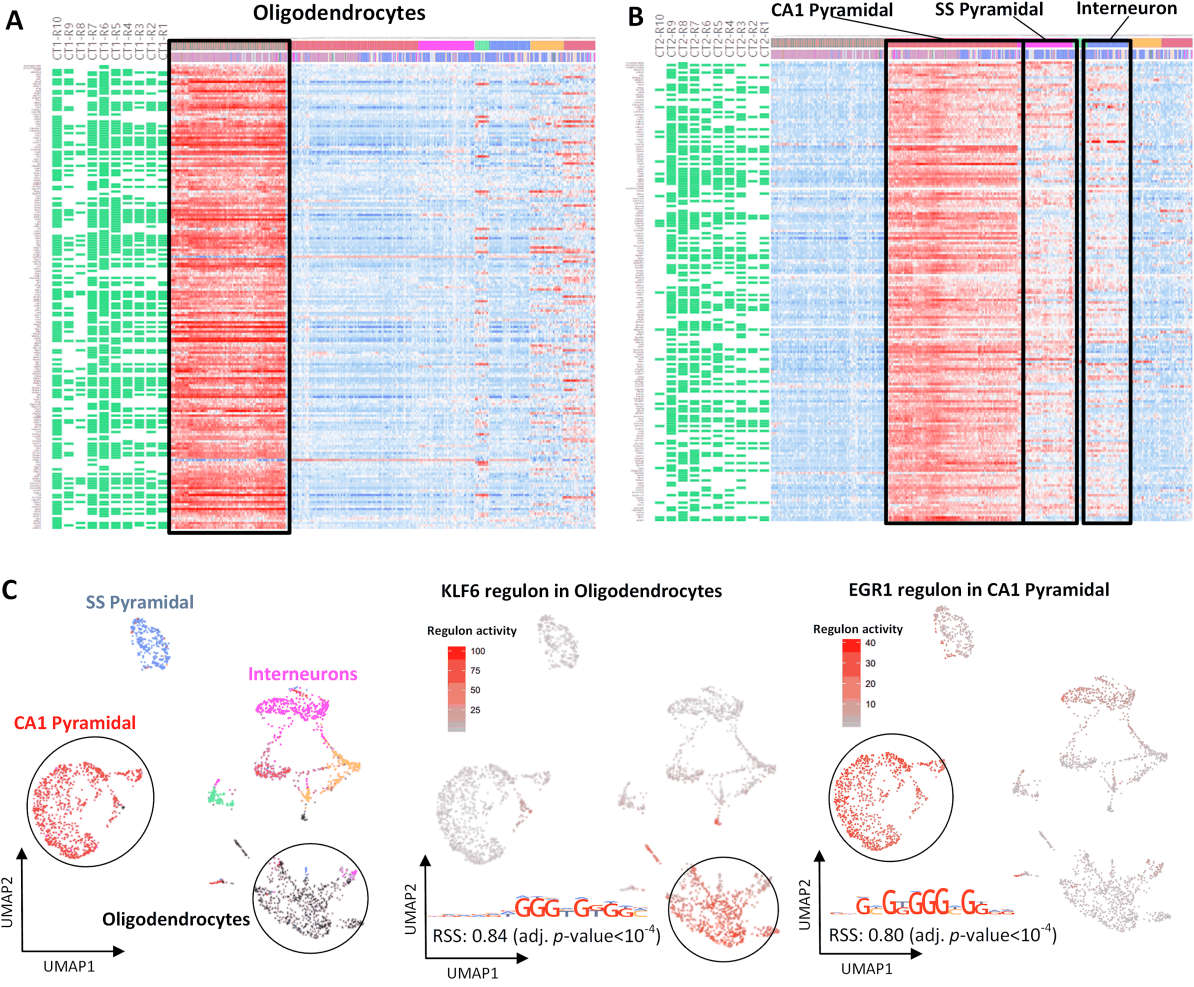
CTSRs identified by IRIS3 can elucidate the characteristics of mouse neuron cell types. (A) Gene conformations and expression heatmap of the top ten regulons (all are CTSRs) identified in oligodendrocyte lineage cells, including OPCs. (**B**) Gene conformations and expression heatmap of the top ten regulons (all are CTSRs) identified in CA1 Pyramidal. Most of these genes are also highly expressed in SS Pyramidal and Interneurons (**C**) UMAP plots colored by cell types contrasts to the regulon UMAP of KLF6 and EGR1 regulons in oligodendrocyte lineage cells and CA1 Pyramidal, respectively.

We reasoned that CTSRs can be used to reliably characterize and distinguish cell types, and would have functional relevance in these cell types. To illustrate the functional relevance using two examples, all regulons identified in (i) oligodendrocyte lineage cells and (ii) CA1 pyramidal cells are CTSRs, and their co-regulated genes display significantly higher expression values compared to other cell types (Figure 5A, B). TFs and genes included in the oligodendrocyte lineage and CA1 pyramidal cell CTSRs are functionally related to each cell type and can be validated from the literature.

#### Example (i): oligodendrocyte lineage

Sensory enrichment triggers oligodendrocyte progenitor cells (OPCs) to differentiate into myelinating oligodendrocytes the mature somatosensory cortex, which accelerates information transfer in these circuits (49,50). KLF6 is a key TF associated with OPC differentiation, indeed OPCs in mice with lineage-selective KLF6 inactivation undergo maturation arrest followed by apoptosis, and myelination of axons fails (51). In line with this, we identified a major CTSR within CT1 (oligodendrocyte lineage, including OPCs) that is controlled by KLF6 (CT1-R2 RSS = 0.84) (51) (Figure 5C).

#### Example (ii): CA1 pyramidal neurons

Hippocampal long-term potentiation (LTP) is an activity-dependent process that provides a means for learning and memory storage, by causing causes long-term increases in synaptic strength between neurons (52). To understand the TFs required for gene transcription and translation during LTP, Chen *et al.* used RiboTag technology to exclusively label excitatory CA1–3 pyramidal neurons (53). The authors found significant upregulation of the transcription factors EGR1 and STAT1 by pyramidal neurons during LTP (53). Consistent with this, we identified CTSRs within CT2 (CA1 pyramidal neurons) tightly controlled by EGR1 (CT2-R5 RSS = 0.80) (Figure 5C) and STAT1 (CT2-R16 RSS = 0.76).

Finally, we also aimed to match our data against ATAC-seq data. 51 out of 90 (56.6%) target genes in the KLF4 regulon in oligodendrocyte lineage cells (important for the early stages of OPC differentiation) (54), were matched to the same cranial neural crest ATAC-Seq data. This enrichment test provides a way to validate the TF-gene interactions in a CTSR, though the matching rate is moderate due to the low specificity of bulk ATAC-Seq data.

## CONCLUDING REMARKS

IRIS3 is not a static server, but is highly amenable to continuous improvements to increase the accuracy and efficiency of CTSR inference. Indeed, current limitations of IRIS3 that we aim to improve in future updates include: (i) Gene imputation may induce false positives when applied to data with highly variable distributions. To alleviate this issue, some studies have been carried out to integrate bulk RNA-Seq data to correct dropouts in scRNA seq data (55,56). In the future IRIS3 updates, we will recover expression estimates from scRNA-Seq data via iteratively integrating cell-type-specific co-expressed gene modules in a bulk RNA-Seq deconvolution framework. (ii) Theoretically, features retained in biclusters characterize the signals of cells. Hence, the biclusters could be used for a simultaneous prediction of cell types and inference of cell-type-specific gene modules, rather than the current gene module assignment step in IRIS3. (iii) Smart-Seq2 data and 10× Genomics data have their respective advantages in deciphering cell heterogeneity. Smart-Seq2 data includes fewer cells and higher read depth that can be used for recognizing gene expression patterns and capturing accurate DEGs; and 10× Genomics data includes more cells but lower read depth, which is valuable for identifying major cell types. The integration analysis of scRNA-Seq data from different sequencing technologies can potentially contribute to accurate CTSR identification. (iv) The joint analysis of single-cell multi-omics data (i.e. matched scRNA-Seq and scATAC-Seq) presents us with an unprecedented opportunity to build TF-gene linkages (57), however, identifying the significant correlations between scATAC-Seq peaks and scRNA-Seq genes is a non-trivial task.

Overall, IRIS3 is a highly effective and easy-to-use web server for biologically meaningful CTSR inference. CTSRs inferred and validated by IRIS3 can provide a finer characterization of complex regulatory mechanisms in diverse cell types. The power and convenience of IRIS3 are further enhanced by its ability to be integrated with other computational or experimental tools for important biomedical fields, including mutation detection in complex diseases, tumor hierarchies and heterogeneity, causal gene regulatory network construction, and drug development.

## IMPLEMENTATION

IRIS3 runs on a Red Hat Enterprise seven Linux system with 28-core Intel Xeon E5–2650 CPU and 64GB RAM, and each task is assigned to four cores and scalable based on the server load. The front-end builds on top of technologies such as JQuery and Bootstrap, the interactive tables and figures are generated utilizing libraries such as DataTables, Plotly.js, and Clustergrammer (58). We employed PHP for the back-end server implementation, and the data parser workflow is aggregated using the R programming language. All data are stored and managed using a MySQL database.

## AVAILABILITY OF DATA AND MATERIALS

IRIS3 is an open-source web server freely available from https://bmbl.bmi.osumc.edu/iris3/ without login requirement. The source code is available at https://github.com/OSU-BMBL/IRIS3. All data can be downloaded from the IRIS3 server, and the source data can be retrieved from Gene Expression Omnibus and European Bioinformatics Institute databases using the data ID listed in Supplementary Table S3.

## Supplementary Material

gkaa394_Supplemental_Files
