# Supplementary material for: IRIS3: integrated cell-type-specific regulon inference server from single-cell RNA-Seq
Source: Nucleic Acids Res. 2020 May 18;48(W1):W275–86. doi: 10.1093/nar/gkaa394 (PMC7319566; doi:10.1093/nar/gkaa394)
Supplement: gkaa394_Supplemental_Files [file gkaa394_supplemental_files.zip › Supplementary-Information-04052020.docx]

**Supplementary DATA for**

**IRIS3: Integrated Cell-type-specific Regulon Inference Server from Single-cell RNA-Seq**

Anjun Ma^1,*^, Cankun Wang^1,*^, Yuzhou Chang^1^, Adam McDermaid^2,3^, Bingqiang Liu^4^, Chi Zhang^5^, and Qin Ma^1,$^

^1^ Department of Biomedical Informatics, College of Medicine, The Ohio State University, OH, 43210, USA,

^2^ Imagenetics, Sanford Health, SD, 57104, USA,

^3^ Department of Internal Medicine, Sanford School of Medicine, University of South Dakota, SD, 57069, USA,

^4^ School of Mathematics, Shandong University, Jinan, 250100, China,

^5^ Department of Medical & Molecular Genetics, Indiana University, School of Medicine, Indianapolis, IN, 46202, USA

* These authors contributed equally to the paper as first authors.

^$^ To whom correspondence should be addressed. Dr. Qin Ma. Tel: (706)-254-4293; Email: [qin.ma@osumc.edu](mailto:qin.ma@osumc.edu)

Supporting Website: <https://bmbl.bmi.osumc.edu/iris3/>

Software Tutorial: <https://bmbl.bmi.osumc.edu/iris3/tutorial.php>

Software FAQ: <https://bmbl.bmi.osumc.edu/iris3/more.php>

Supplementary Figure S1: IRIS3 job submission page.

Supplementary Figure S2: RSS significance test.

Supplementary Figure S3: Additional results of the example dataset.

Supplementary Methods

Method S1. QUBIC2 and gene module detection.

Method S2. Hypergeometric test for determining active gene modules.

Method S3. DMINDA2.0.

Method S4. MEME.

Method S5. RAS and RSS calculation.

Method S6. Index evaluations for cell type prediction.

Method S7. Log-transformed expression value for heatmap displaying.

Method S8. ATAC-Seq enrichment validation.

Method S9. TAD supported supplementary gene finding**.**

Supplementary Table S1: IRIS parameter settings.

Supplementary Table S2: Integrated tools, packages, and databases in IRIS3.

Supplementary Table S3: Information of tested data.

Supplementary Table S4: Performance comparison between IRIS3 and SCENIC.

**Supplementary Figure S1**: IRIS3 job submission page. (1) Example data can be chosen or downloaded from the drop-down selections. (2) Upload your expression file(s) by selecting or dragging the file from local to the drop-zone. Note: The maximum number of upload files is 3, typically designed for your 10X genomics raw gene-barcodes matrix files. Note that do not unzip your files, otherwise, our server will consider you uploading a text format gene expression file and will try to display the preview. (3) Choose the species. (4) Advanced options. (5) An email is not required to submit the job but highly recommended. (6) Submit the job once everything is ready. (7) Check our example result page. (8) Select whether to carry out the gene imputation step. (9) Change clustering parameters in Seurat. (10) Change biclustering parameters in QUBIC2. (11) Change the upstream promoter region. (12) Select either quick mode or default mode. (13) Provide cell labels for CTSR identification. (14) Upload additional gene modules.

**Supplementary Figure S2**: RSS significance test. The number of random genes was set to 40 considering the average gene number in all active regulons we tested. N was set to 10,000 times. For any active regulons, if its RSS is greater than 95% of the random RSSs in the corresponding cell type (p-value<0.05), we consider it as a CTSR. Active regulons with small RSS can still be significant if the overall gene expression within the cell type is low.

**Supplementary Figure S3**: Additional results of the example dataset. (A) The results of cell type prediction. Four index scores are used to evaluate the closeness of the predicted cell type to the benchmark. Two UMAPs show the benchmark cell label and predicted cell clusters, respectively. (B) A trajectory plot is generated from Slingshot integrated with IRIS3. (C) The silhouette plot and Sankey plot are used to evaluate the accuracy of cell type prediction. Benchmarked cell labels are required to generate the index scores, benchmark cell type UMAP, and the Sankey plot. (D) A complete marker gene list generated from differential expression analysis using Seurat. (E) Pre-processing results, number of cells, number of active regulons per cell type, and the records of job settings.

**Supplementary Methods**

**Method S1. QUBIC2 and gene module detection**. Qualitative Biclustering Version 2 (QUBIC2) is a biclustering algorithm that simultaneously clusters by rows and columns, generating local low-rank sub-matrices from the input expression matrix. This tool utilizes a qualitative (or semi-qualitative) measurement of expression, along with combinatorial optimizations, to generate a representative matrix(1). QUBIC2 also implements a weighted graph to present the representative matrix in terms of the similarity of every pair of genes. The weighted graph provides genes as vertices, edges as connections between every two pairs of genes, and weights being the similarity between two entire rows, with higher weights indicating higher similarity. This weighted graph procedure is used to define the series of biclusters. Overlap level controls the level (from 0 to 1) of overlaps between the to-be-identified biclusters. 0 means no overlap and 1 indicates complete overlap. The default value is 0.7. Max biclusters control the maximum number of to-be-identified biclusters. A larger number may sufficiently increase the running time and induce noise as more fragmental biclusters generated. The default value is 500.

**User’s gene module analysis.** For those users who have their own gene modules, either collected from literature or generated from other tools, they can upload the gene lists (modules) to IRIS3 as an option for regulon inference. Those regulons will be regarded as “module-specific regulons” and have individual tabs next to the last cell type to show the heatmap and results. In addition to the similar CTSR finding function described below, users can compare CTSRs of interest with the regulons referred from the self-uploaded gene modules and find any similarities.

**Method S2. Hypergeometric test for determining active gene modules**. The probability of having $x$ cells of the same cell type in a bicluster of size $n$ from the dataset with a total of $N$ cells can be computed using the following hypergeometric function:

$$P(X=x\text{|}N,p,n)={\left( \begin{matrix} pN \\ x \end{matrix} \right)\left( \begin{matrix} \left( 1-p \right)N \\ n-x \end{matrix} \right)}/\left( \begin{matrix} N \\ n \end{matrix} \right)$$

where $p$ is the percentage of that cell type among all cell types in the dataset. The *p*-value of getting such enriched bicluster is calculated as:

$$p\text{-}value=P\left( X\geq x \right)=1-P\left( X-x \right)=1-\sum_{i=0}^{x-1} \frac{\left( \begin{matrix} pN \\ i \end{matrix} \right)\left( \begin{matrix} \left( 1-p \right)N \\ n-i \end{matrix} \right)}{\left( \begin{matrix} N \\ n \end{matrix} \right)}$$

Adjusted *p*-value is calculated as:

$$adj.p\text{-}value=p\text{-}value\times N_{cell type}\times N_{bicluster}$$

where $N_{cell type}$ denotes to the number of cell types and $N_{bicluster}$ denotes to the total number of biclusters. A bicluster is considered to be active in the corresponding cell type if the cell hypergeometric result is significant (adj.*p*-value<0.05)

**Method S3. DMINDA2.0**. The second version of the DNA Motif Identification and Analysis server (DMINDA2.0) is an integrated web server for motif identification and analyses(2,3). Included in this server are six functionalities: motif finding, motif scanning, motif comparison, motif co-occurrence analysis, motif prediction, and regulon prediction. The former three of these functionalities are utilized within IRIS3. Given a set of promoters for genomic sequences, DMINDA2.0 utilizes the BOBRO algorithm to identify statistically significant motifs by searching for these short-conserved sequences while avoiding the background noise. If provided with a query motif, DMINDA2.0 also scans provided sequences for all motif instances. Finally, DMINDA2.0 provides comparisons for the similarity of a set of query motifs and performs clustering of similar motifs.

**Method S4. MEME**. The Multiple EM for Motif Elicitation (MEME) suite is capable of performing motif finding, scanning, and comparisons(4). The GLAM2 algorithm, which is specifically developed to identify gapped motifs, is used for *de-novo* motif finding. MAST, FIMO, and GLAM2SCAN are used by the MEME suite to scan sequences for provided query motifs or those identified by the GLAM2 algorithm. Identified motifs can also be compared against motif databases using the TOMTOM algorithm to scan popular motif databases.

**Method S5. RAS and RSS calculation**. For each active regulon, the RAS in a cell is calculated using the Wilcoxon rank sum test (using wmwTest function in the BioQC R package (5)) to rank the expression value of genes in the regulon to the rest genes. This method tends to generate more CTSRs and is more efficient than the AUCell method used in SCENIC, without any dependency on thresholds. The procedure of calculating the specificity of an active regulon under one cell type is adopted based on an entropy-based strategy (6) which is previously used for gene expression data analysis. For each active regulon, we use a vector to represent the distribution of RAS in the cell population:

$$P^{R}= \left( p_{1}^{R}, \cdots,p_{n}^{R} \right)$$

where $n$ is the total number of the cells. We assign a weight of 2 to the RAS if the cell belongs to the specific cell type:

$$p_{i}^{R}=\left\{ \begin{aligned} 2 \times p_{i}^{R},cell belongs to the specific cell type \\ p_{i}^{R}, otherwise \end{aligned} \right.$$

and RAS are normalized so that:

$$\sum_{i=1}^{n} p_{i}^{R}=1$$

Then we used a vector to indicate whether a cell belongs to a specific cell type:

$$P^{C}= \left( p_{1}^{C}, \cdots,p_{n}^{C} \right)$$

where

$$P_{i}^{C}=\left\{ \begin{aligned} 1, cell belongs to the specific cell type \\ 0, otherwise \end{aligned} \right.$$

The vector is also normalized so that:

$$\sum_{i=1}^{n} p_{i}^{C}=1$$

Next, we evaluate the Jensen-Shannon Divergence (JSD), which is a commonly used metric for quantifying the difference between two probability distributions, defined as:

$$JSD\left( P^{R}{,P}^{C} \right)=H\left( \frac{P^{R}+P^{C}}{2} \right)-\frac{H\left( P^{R} \right)+H(P^{C})}{2}$$

where $H\left( P \right)=-\sum p_{i}\log p_{i}$ represents the Shannon entropy of a probability distribution $P$. The range of JSD values is between 0 and 1, where 0 means identical distribution and 1 means extreme difference. Finally, the RSS is defined by converting JSD to a similarity score:

$$RSS\left( R,C \right)=1-\sqrt{JSD\left( P^{R}{,P}^{C} \right)}$$

To calculate the empirical p-value of an RSS in one cell type, we adapt a bootstrap simulation strategy:

$${RSS\left( R,C \right)}_{p\text{-}value}=\frac{\sum(RSS\left( sim \right)\leq RSS\left( R,C \right))}{N}$$

where the $RSS\left( sim \right)$ is the RSS calculated as above using random genes. The number of randomly selected genes was set to 40 considering the average gene number in all active regulons we tested. N was set to 10,000 times. Regulon *p*-values are Bonferroni-adjusted by multiplying the number of regulons in the exact cell type. Regulons with adjusted *p*-values less than 0.05 (by default) are considered CTSRs. All active regulons and CTSRs are ranked and rearranged by the RSS p-value in ascending order so that the most significant CTSR will be displayed in the first place.

**Method S6. Index evaluations for cell type prediction**. Four index scores, Rand Index (RI), Adjusted Rand Index (ARI), Fowlkes and Mallows's index (FMI), and Jaccard Index (JI) are calculated to evaluate the similarity between the prediction and ground-truth labels. A Silhouette score showing how similar each cell is to its type compared to other clusters is also calculated. If the ground-truth cell type file is not provided by users, both the index scores and Silhouette scores are omitted since no comparing target can be accessed.

The index scores can be used to calculate the similarity between the predicted cell labels and validated experimental labels (if provided), and a higher index score indicates a higher accurate prediction. Given two objects, A and B, each with n binary attributes. The indices are useful measures of the overlap that A and B share with their attributes. The index score ranges from 0 to 1. The total number of each combination of attributes for both A and B are specified as follows:

- $TP$*:* the total number of attributes where A and B both have a value of 1.
- $TN$: the total number of attributes where A and B both have a value of 0.
- $FP$: the total number of attributes where the attribute of A is 1 and the of B is 0.
- $FN$: the total number of attributes where the attribute of A is 0 and the of B is 1.

$RI$ can be calculated as:

$$RI=\frac{TP+TN}{TP+TN+FP+FN}=\frac{TP+TN}{\left( \begin{matrix} n \\ 2 \end{matrix} \right)}$$

$ARI$ is the corrected-for-chance of the $RI$. To calculate $ARI$, a contingency table is built to summarize the overlaps between the two cell label lists with n elements (cells). Each entry denotes the number of objects in common between the two label lists. The $ARI$ score can be calculated as:

$$ARI=\frac{\frac{\sum_{ij} \binom{n_{ij}}{2}-\left[ \sum_{j} \binom{a_{i}}{2}\sum_{j} \binom{b_{j}}{2} \right]}{\binom{n}{2}}}{\frac{1}{2}\left[ \sum_{i} \binom{a_{i}}{2}+\sum_{j} \binom{b_{j}}{2} \right]-\frac{\left[ \sum_{i} \binom{a_{i}}{2}\sum_{j} \binom{b_{j}}{2} \right]}{\binom{n}{2}}}$$

where $n_{ij}$ are values from the contingency table, a_i_ is the sum of the $i$th row of the contingency table, $b_{j}$ is the sum of the $j$th column of the contingency table.

Two more common index criteria, $FMI,$ and $JI$, also provide the evaluation of similarity between the two cell type lists, and can be calculated by the following equations, respectively:

$$FMI=\sqrt{\frac{TP}{TP+FP}\times\frac{TP}{TP+FN}}$$

$$JI=\frac{TP}{TP+FP+FN}$$

Silhouette refers to a method of interpretation and validation of consistency within clusters of data. The technique provides a succinct graphical representation of how well each object l cluster. The silhouette value is a measure of how similar an object is to its luster (cohesion) compared to other clusters (separation). The silhouette ranges from −1 to +1, where a high value indicates that the object is well matched to its cluster and poorly matched to neighboring clusters. The silhouette score can be calculated by:

$$s\left( i \right)=\frac{b\left( i \right)-a(i)}{max\left\{ a\left( i \right),b(i) \right\}}=\left\{ \begin{aligned} 1-\frac{a\left( i \right)}{b\left( i \right)}, if a\left( i \right)<b(i) \\ 0, if a\left( i \right)=b(i) \\ \frac{b\left( i \right)}{a\left( i \right)}-1, if a\left( i \right)>b(i) \end{aligned} \right.$$

where $a(i)$ be the average distance between a sample i and all the rest samples in the same cluster, and $b\left( i \right)$ be the smallest average distance of $i$ to all samples.

**Method S7. Log-transformed expression value for heatmap displaying.** To better illustrate the expression value of the gene sets, we applied a log-transformed for heatmap interpretation:

$${VN}_{i}=\lg\left( 1+{VE}_{i} \right)-\sum_{i=1}^{n} \lg\left( 1+{VE}_{i} \right)/n$$

where ${VN}_{i}$ indicates the normalized value for gene$i$, ${VE}_{i}$indicates the expression value of gene $i$, and n is the number of total cells.

**Method S8. ATAC-Seq enrichment validation.** Genes can be regulated only if their promoter regions are located in the open chromatin area that is available for the regulator to bind. We downloaded several ATAC-Seq data of human (74 tissue) and mouse from CistromeDB (<http://cistrome.org/db/#/>)(7). Specifically, we compare all motifs in a CTSR to the peak regions from the ATAC-Seq. If a motif instance overlaps with the peak region, it will be considered as an open chromatin region enriched motif which is available for the binding of the corresponding TF. Genes in the CTSR and supported by the ATAC-Seq are then validated as a true target gene. However, such chromatin activities (peaks) are diverse in different cell types or even cells. The bulk ATAC-Seq data we found for different tissues/cell types cannot provide accurate validation to target genes. Thus, such a function is only additionally provided to users to have a general glance. In the future, we are expecting to have single-cell ATAC-Seq data pairing with scRNA-Seq data for credible gene validations in CTSRs.

**Method S9. TAD supported supplementary gene finding.** Considering the computational efficiency, the region for motif finding in IRIS3 is limited to 1000-2000 bp region upstream to each gene, and motifs outside of the region may not be found. To amend such a deficiency, we integrated TAD data resulted from Hi-C to trace back genes in the active gene modules of a cell type but not in the CTSR. Genes in the same TAD are more likely to be regulated together due to the topological folding structure of such a region. We first downloaded 19 human tissue and five mouse tissues TAD files, respectively, and identified all genes for each TAD region per file. TAD regions are selected if any genes in a CTSR are found in the TAD gene list. The remaining TAD supported genes are used to search in the active gene modules of that CTSR and output all matched genes as TAD supplementary genes.

**References**

1. Xie, J., Ma, A., Zhang, Y., Liu, B., Cao, S., Wang, C., Xu, J., Zhang, C. and Ma, Q. (2020) QUBIC2: a novel and robust biclustering algorithm for analyses and interpretation of large-scale RNA-Seq data. *Bioinformatics*, **36**, 1143-1149.

2. Ma, Q., Zhang, H., Mao, X., Zhou, C., Liu, B., Chen, X. and Xu, Y. (2014) DMINDA: an integrated web server for DNA motif identification and analyses. *Nucleic Acids Res*, **42**, W12-19.

3. Yang, J., Chen, X., McDermaid, A. and Ma, Q. (2017) DMINDA 2.0: integrated and systematic views of regulatory DNA motif identification and analyses. *Bioinformatics*, **33**, 2586-2588.

4. Bailey, T.L., Boden, M., Buske, F.A., Frith, M., Grant, C.E., Clementi, L., Ren, J., Li, W.W. and Noble, W.S. (2009) MEME SUITE: tools for motif discovery and searching. *Nucleic Acids Res*, **37**, W202-208.

5. Zhang, J.D., Hatje, K., Sturm, G., Broger, C., Ebeling, M., Burtin, M., Terzi, F., Pomposiello, S.I. and Badi, L. (2017) Detect tissue heterogeneity in gene expression data with BioQC. *BMC Genomics*, **18**, 277.

6. Cabili, M.N., Trapnell, C., Goff, L., Koziol, M., Tazon-Vega, B., Regev, A. and Rinn, J.L. (2011) Integrative annotation of human large intergenic noncoding RNAs reveals global properties and specific subclasses. *Genes Dev*, **25**, 1915-1927.

7. Zheng, R., Wan, C., Mei, S., Qin, Q., Wu, Q., Sun, H., Chen, C.H., Brown, M., Zhang, X., Meyer, C.A. *et al.* (2019) Cistrome Data Browser: expanded datasets and new tools for gene regulatory analysis. *Nucleic Acids Res*, **47**, D729-D735.
